# Current vaccination status and safety of children with peripheral neuroblastoma in the real-world

**DOI:** 10.3389/fimmu.2023.1278258

**Published:** 2024-01-08

**Authors:** Heping Shen, Yuyang Xu, Yuxuan Zhan, Yan Liu, Xuechao Zhang, Mingyan Li, Chai Ji

**Affiliations:** ^1^ Department of Pediatric Hematology, Children’s Hospital, Zhejiang University School of Medicine, Hangzhou, China; ^2^ Department of Expanded Program on Immunization, Hangzhou Center for Disease Control and Prevention, Hangzhou, Zhejiang, China; ^3^ Public Health, Zhejiang University, Hangzhou, China; ^4^ Department of Pediatric Health Care, Children’s Hospital, Zhejiang University School of Medicine, Hangzhou, China

**Keywords:** peripheral neuroblastoma, children, immunization rates, security, immunization program

## Abstract

**Background:**

peripheral neuroblastic tumors (pNT) have high incidence and mortality, and infants are prone to various infectious diseases. The purpose of this study is to understand the immunization status of children with pNT in the real-world and the incidence of adverse reactions after vaccination, and to evaluate the feasibility of vaccination and the influencing factors of vaccination.

**Methods:**

Children with pNT treated in the Children’s Hospital Affiliated to Zhejiang University from January 1, 2011 to December 1, 2021 were included. By referring to medical records, the vaccination history of the national immunization program (NIP) vaccines and the occurrence of adverse events following immunization(AEFI), current status and safety of immunization in children with pNT in the real-world were analyzed.

**Results:**

Among 784 children with pNT, 394 were able to obtain the history of vaccination. The overall vaccination rate of NIP vaccines was 71.49% before chemotherapy and 37.67% after chemotherapy, and the recovery time of vaccination after treatment was 16.00 (6.00,24.00) months. Age, time of tumor diagnosis and disease classification were significantly correlated with vaccination. AEFI reported an incidence of 0.23‰.

**Conclusion:**

The vaccination rate of children with pNT is generally low, especially the vaccination rate after chemotherapy. The vaccination safety is good, children should be encouraged to immunize.

## 1 Introduction

Cancer is the second leading cause of death among children aged 5 to 14 years after accidents, and more than one third of children diagnosed with tumors are before the age of five (1). In 2020, a retrospective survey of 938 children in Beijing over the past decade found that the three most common types of infant malignant solid tumors were retinoblastoma (39%), neuroblastoma (28.4%) and hepatoblastoma (14.2%) (2). Neuroblastoma is one of peripheral neuroblastic tumors (pNT). The International Neuroblastoma Pathology Classification has developed the classification of pNT (3), including 4 types of tumors: 1) neuroblastoma (NB); 2) ganglio-neuroblastomainter mixed (GNBi); 3) ganglioneuroma (GN); 4) ganglio-neuroblastomanodular (GNBn), the first 3 types representing the maturation process of NB, they are rare benign tumors (4). The last type is polyclonal, the differentiation degree of GNB is between NB and GN, and the malignancy degree is lower than NB (5).

All ganglioneuromas used to be NB in early development, and ganglioneuromas are rare in infancy compared to older children, so NB is the most common and deadly tumor in pNT (6). If children were diagnosed to be in the late stage of NB, the prognosis is poor (7). It accounts for 8-10% of all childhood cancer cases, with the highest incidence occurring in newborns under one year of age, 80.8% in children under five years of age and only 1.6% in children over 10 years of age, with more males than females (8). In the United States, the incidence of NB is 11-13 cases per million children under 15 years of age (9); In China, the incidence of NB among live births is about 7.7 cases per million (10).

Children with tumor have great damage to their immune ability due to the disease itself and chemotherapy (11–13). Studies have shown that the prognosis of high-risk children is much worse than that of low-risk and medium-risk children, and the poor prognosis is closely related to low immune capacity (14 15).This is because high-risk neuroblastoma tumors are characterized by a low number of immune cells in the tumor microenvironment and are commonly referred to as “cold” tumors (14). Patients with neuroblastoma typically present with an impaired immune system at the time of diagnosis, and while chemotherapy can lead to an initial increase in total white blood cell and lymphocyte counts (15), further aggressive treatment can suppress immune function (16). Consequently, it can result in the disruption of both humoral and cellular immunity in children with neuroblastoma (17). In terms of humoral immunity, there is a decrease in levels of immunoglobulins IgG, IgA, IgM, and IgE. Regarding cellular immunity, there is a reduction in total T lymphocytes (TTL) as well as in the CD4+/CD8+ ratio compared to levels prior to chemotherapy (18). After therapy in the CD8 T cell compartment, signs of immune reconstitution were observed five years after diagnosis and treatment (19). However, no immune reconstitution was observed with autologous hematopoietic stem cell transplantation and increased immunotherapy (20). Therefore, the child’s immune system is weak after diagnosis and treatment, which can lead to a high rate of infection (21, 22). Study have found that NB patients have a high burden of infection, with a cumulative incidence of 45% during treatment, and an infection rate (IR) of 0.19/100 patient-days-at risk (23).

In recent years, the vaccination coverage rate of children under the National Immunization Program (NIP) in China has reached over 90% (24), the vaccination rate of children under the NIP is good. However, a survey of children receiving medical treatment in Guangzhou showed that 87.8% of them were delayed due to special diseases (25), the vaccination rate among children with tumors and other malignant diseases was significantly lower than that of normal children (26, 27), the completion rate of planned immunization is low.

Immunization, an essential approach to bolster immune function and reduce infections, also plays a significant role in enhancing the prognosis of immune levels in children with tumors (28, 29). Research has demonstrated a low occurrence of adverse reactions among vaccinated children (30), including those diagnosed with neuroblastoma (31, 32). By collecting and sorting disease data and immunization data of pNT children after chemotherapy, this study analyzed the current immunization status of children and the occurrence of adverse reactions by using the analysis method of status study, and obtained the immunization coverage rate and incidence of adverse reactions of pNT children, providing suggestions and guidance for pNT children immunization.

## 2 Materials and methods

### 2.1 Study design

A retrospective study was conducted on children diagnosed as pNT and treated in Children’s Hospital of Zhejiang University from January 1, 2011 to December 1, 2021.

### 2.2 Participants

A total of 784 children who had been diagnosed with peripheral neuroblastoma by oncologists and who had received treatment were included. Exclusion criteria included patients with other systemic malignancies and patients over 16 years of age at first diagnosis. The pNT classification includes four types of tumors: 1) NB; 2) GNBi; 3) GN; 4) GNBn. The children in the study were categorized as either belonging to Hangzhou or non-Hangzhou. Additionally, the age of the children was grouped into four categories: 1-5 years, 5-10 years, 10-15 years, and 15+ years, with each group separated by a five-year interval. The tumor diagnosis age was the child’s first admission to the hospital for chemotherapy, while whose actual age was determined from their year of birth to July 2022. Lastly, the vaccination recovery time refers to the duration between the first vaccination after completing chemotherapy.

### 2.3 Data collection

The patient’s clinical and basic information is collected from the hospital’s tumor reporting system, including the patient’s name, sex, age, household registration, date of hospitalization, hospitalization number, disease diagnosis and treatment history. The vaccination records of target participants were extracted from the Zhejiang Provincial immune Information System. Data on Abnormal Reactions to Vaccination (AEFI) were collected from the China National Adverse Event Information System for Immunization.

In this study, the Immunization records collected included 13 types of NIP vaccines with 28 doses each (**Table 1**). Replacement vaccinations are also equivalent to vaccination completion.

**Table 1:** National Immunization Program Vaccine Immunization Schedule for Children (2021 version).

Vaccines	Recommended age for vaccination													
	at birth	1 month	2 months	3 months	4 months	5 months	6 months	8 months	9 months	18 months	2 years	3 years	4 years	6 years
HepB	1^st^ dose	2^nd^ dose					3^rd^ dose							
BCG	1^st^ dose													
IPV			1^st^ dose	2^nd^ dose										
bOPV					1^st^ dose								2^nd^ dose	
DTaP				1^st^ dose	2^nd^ dose	3^rd^ dose				4^th^ dose				
DT														one dose
MMR								1^st^ dose		2^nd^ dose				
JE-L								1^st^ dose			2^nd^ dose			
JE-I								1^st^ and 2^nd^ dose			3^rd^ dose			4^th^ dose
MPSV-A							1^st^ dose		2^nd^ dose					
MPSV-AC												1^st^ dose		2^nd^ dose
HepA-L										one dose				
HepA-I										1^st^ dose	2^nd^ dose			

Note: 1. When the live attenuated JE vaccine was selected, two doses of vaccination should be used. When the inactivated JE vaccine was selected, four doses of vaccination were used; The interval between the first and second doses of JE vaccine was 7-10 days.

2. When choosing live attenuated hepatitis A vaccine for vaccination, a one-dose vaccination procedure is used. When selecting the inactivated hepatitis A vaccine, two doses of vaccination are used.

HepB, Hepatitis B vaccine; BCG, Bacillus Calmett-Guerin vaccine; IPV, Inactivated polio vaccine; bOPV, Oral live attenuated polio vaccine; DTaP, Acellular pertussis diphtheria tetanus vaccine; DT, Diphtheria tetanus vaccine; MMR, Measles; mumps, rubella vaccine; JE-L, Live attenuated Japanese encephalitis vaccine; JE-I, Inactivated Japanese encephalitis vaccine; MPSV-A, Meningococcal meningitis-A vaccine; MPSV-AC, Meningococcal meningitis-AC vaccine; HepA-L, Live attenuated Hepatitis A vaccine; HepA-I, Inactivated Hepatitis A vaccine.

Adverse events following immunization (AEFI),including: adverse reactions (including general reactions and abnormal reactions), vaccine quality incidents, vaccination incidents, coincidences, and psychogenic reactions.

### 2.4 Correlation index calculation


$$\begin{array}{c} \text{Vaccination coverage}\\ =\frac{\text{The number of people vaccinated in a study population over a given period of time }}{\text{The population of the study population at the same time}}\times 100\% \end{array}$$

$$\begin{array}{c} \mathrm{Incidence}\;\mathrm{of}\;\mathrm{adverse}\;\mathrm{reactions}\\ =\frac{\text{The number of adverse reactions occurring in the vaccinated population over a given period of time }}{\text{Total number of vaccination doses in the same time period}}\times 100\% \end{array}$$

### 2.5 Statistical analysis

Basic Excel software was used for data screening and sorting, and then SPSS 25.0 software was used for data analysis. Normal measurement data were represented by (X ± S), T-test was used for comparison between two groups, and analysis of variance was used for comparison between multiple groups. Non-normal measurement data were represented by M (P_25_,P_75_), and rank sum test was used for comparison between groups. The rate or percentage of counting data was expressed, and the chi-square test was used for comparison between groups. Logistic regression model was used to analyze the influencing factors. *P*<0.05 was considered statistically significant.

In the analysis, according to the age distribution, the actual age was analyzed as a group of 5 years, while the tumor diagnosis age was analyzed as a group of 36 months, for the median age at tumor diagnosis was 34 months (7).

### 2.6 Ethical considerations

This project was approved by the Ethics Committee of Hangzhou Center for Disease Control and Prevention (judgment reference number: 2021-18) and the Ethics Committee of the School of Medicine, Children’s Hospital of Zhejiang University (judgment number: 2022-IRB-028).

## 3 Results

### 3.1 Demographic characteristics

A total of 394 children from 784 were given vaccination data. According to the inoculation data, 52.79% (208/394) were males and 47.21% (186/394) were females. The local population accounted for 31.22% (123/394) and the non-local population accounted for 68.78% (271/394). 53.81% of the patients were 5-10 years old; 45.43% of the patients were younger than 36 months of age; 81.98% of the cases were NB, 16.50% GNBi and 1.52% GN (**Table 2**).

**Table 2:** Sample characteristics (N=394).

Characteristics	N	%
Gender		
Male	208	52.79
Female	186	47.21
Location		
Hangzhou	123	31.22
non-Hangzhou	271	68.78
Actual age (year)		
1-5	109	27.66
6-10	212	53.81
11-15	62	15.74
16-	11	2.79
Tumor diagnosis age (month)		
0-36	179	45.43
37-72	147	37.31
73-108	39	9.90
109-	25	6.35
Pathological classification		
NB	323	81.98
GNBi	65	16.50
GNBn	0	0.00
GN	6	1.52
Total	394	100

NB, neuroblastoma; GNBi, ganglio-neuroblastomainter mixed; GN, ganglioneuroma; GNBn, ganglio-neuroblastomanodular.

### 3.2 Vaccination status of NIP vaccines

The coverage rates of the first, second and third doses of HepB vaccine were 95.18%, 93.40% and 78.93% before chemotherapy, and were 10.53%, 23.08% and 54.22% after chemotherapy; the coverage rate of BCG vaccine was 91.62% before chemotherapy, and was 21.21% after chemotherapy, the coverage rates of the first and second doses of IPV vaccine were 91.37% and 88.07% before chemotherapy, and were 38.24% and 38.30% after chemotherapy; the coverage rates of the first and second doses of bOPV vaccine were 83.76% and 46.51% before chemotherapy, and were 46.88% and 27.54% after chemotherapy. The vaccination rates of the first, second, third and fourth doses of DTaP vaccine were 87.82%, 86.29%, 84.77% and 66.49% before chemotherapy, and were 41.67%, 44.44%, 41.67% and 42.97% after chemotherapy. The vaccination rates of DT vaccine was 21.05% before chemotherapy, and was27.56% after chemotherapy; and the vaccination rates of the first and second doses of MMR vaccine were 87.56% and 77.16% before chemotherapy, and were 46.88% and 27.54% after chemotherapy. The vaccination rates of the first and second doses of JE-L vaccine were 72.59% and 60.31% before chemotherapy, and were 50.00% and 36.59% after chemotherapy; the vaccination rates of the first, second, third and fourth doses of JE-I vaccine were 74.37%, 71.32%, 46.31% and 16.14% before chemotherapy, and were 48.51%, 53.10%, 54.37% and 12.50% after chemotherapy; and the vaccination rates of the first and second doses of MPSV-A vaccine were 77.41% and 69.90% before chemotherapy, and were 33.71% and 29.41% after chemotherapy. The coverage rates of the first and second doses of MPSV-AC vaccine were 77.75% and 17.89% before chemotherapy, and were 29.41% and 63.64% after chemotherapy. HepA-L vaccine was 63.61% before chemotherapy, and was 43.88% after chemotherapy; and the first and second doses of HepA-I vaccine 66.75% and 63.35%before chemotherapy, and were 7.09% and 37.86% after chemotherapy. The total vaccination rate was 71.49% before chemotherapy, and was 37.67% after chemotherapy. Except that there were no difference in the vaccination rate before and after chemotherapy for DT vaccine, the third and fourth doses of JE-I vaccine, and the vaccination rate after chemotherapy for the second dose of MPSV-AC was higher, the vaccination rate after chemotherapy for other NIP vaccines were significantly lower than that before chemotherapy (**Table 3**).

**Table 3:** Rate of NIP vaccination in children with pNT.

NIP vaccines	NA-b	NS-b	Vaccination rate (%)	NA-a	NS-a	Vaccination rate(%)	χ^2^	*P*
HepB_1_	375	394	95.18	2	19	10.53	152.77	<0.001
HepB_2_	368	394	93.40	6	26	23.08	147.02	<0.001
HepB_3_	311	394	78.93	45	83	54.22	22.12	<0.001
BCG	361	394	91.62	7	33	21.21	120.93	<0.001
IPV_1_	360	394	91.37	13	34	38.24	74.23	<0.001
IPV_2_	347	394	88.07	18	47	38.30	72.93	<0.001
bOPV_1_	330	394	83.76	30	64	46.88	40.75	<0.001
bOPV_2_	60	129	46.51	19	69	27.54	6.71	0.009
DTaP_1_	346	394	87.82	20	48	41.67	64.01	<0.001
DTaP_2_	340	394	86.29	24	54	44.44	54.59	<0.001
DTaP_3_	334	394	84.77	25	60	41.67	58.47	<0.001
DTaP_4_	254	382	66.49	55	128	42.97	22.21	<0.001
DT	60	285	21.05	62	225	27.56	2.93	0.087
MMR_1_	315	394	79.95	30	79	37.97	58.74	<0.001
MMR_2_	256	382	67.02	47	126	37.30	34.75	<0.001
JE-L_1_	286	394	72.59	54	108	50.00	19.78	<0.001
JE-L_2_	237	393	60.31	45	123	36.59	21.26	<0.001
JE-I_1_	293	394	74.37	49	101	48.51	25.16	<0.001
JE-I_2_	281	394	71.32	60	113	53.10	13.24	<0.001
JE-I_3_	182	393	46.31	56	103	54.37	2.12	0.145
JE-I_4_	46	285	16.14	2	16	12.50	0.15	0.699
MPSV-A_1_	305	394	77.41	30	89	33.71	65.24	<0.001
MPSV-A_2_	267	382	69.90	37	115	32.17	52.95	<0.001
MPSV-AC_1_	297	382	77.75	25	85	29.41	75.88	<0.001
MPSV-AC_2_	51	285	17.89	7	11	63.64	14.06	<0.001
HepA-L	243	382	63.61	61	139	43.88	16.32	<0.001
HepA-I_1_	255	382	66.75	9	127	7.09	135.92	<0.001
HepA-I_2_	242	382	63.35	53	140	37.86	27.09	<0.001
Total	7402	10354	71.49	891	2365	37.67	970.25	<0.001

NA-b: the number of children who have actually received the vaccine before chemotherapy; NS-b: the number of children who are compulsory to receive the vaccine before chemotherapy. NA-a: the number of children who have actually received the vaccine after chemotherapy; NS-a: the number of children who are compulsory to receive the vaccine after chemotherapy.

HepB, hepatitis B vaccine; BCG, Bacillus Calmette Guerinvaccine vaccine; IPV, inactivated poliomyelitis vaccine; bOPV, Live Attenuated Oral Poliomyelitis Vaccine; DTaP, Diphtheria and tetanustoxoid with acellular pertussis vaccine; DT, Diphtheria Tetanus vaccine; MMR, Measles mumps and rubella vaccine; JE-L, Live attenuated Japanese encephalitis vaccine; JE-I, Inactivated Japanese Encephalitis vaccine; MPSV-A, Meningococcal polysaccharide vaccineA; MPSV-AC, Meningococcal polysaccharide vaccineAC; HepA-L, live attenuated hepatitis A vaccine; HepA-I, Inactivated HepatitisA vaccine.

Replacement vaccinations are also equivalent to vaccination completion.

### 3.3 The status of re-vaccination after diagnosis and treatment

Among the 394 children, 40.60% (160/394) recovered the immunization of NIP vaccine after chemotherapy, accounting for 52.50% (84/160) in males, 47.50%(76/160) in females and 26.87% (43/160) in Hangzhou, Non-Hangzhou accounted for 73.12% (117/160), the median age of diagnosis was 15.00 (4.30,40.00) months, and the median age of resumption of immunization was 16.0 (6.00,24.00) months (**Table 4**).

**Table 4:** Diagnosis and recovery of the NIP vaccine after treatment.

characteristics	NIP
	N(%)
Gender	
Male	84(52.50)
Female	76(47.50)
Location	
Hangzhou	43(26.87)
non-Hangzhou	117(73.12)
Tumor diagnosis age (month) *	15.00(4.30,40.00)
Resumption of immunization time (month)*	16.00(6.00,24.00)
Total	160(40.60)

*The expression is M (P25,P75).

### 3.4 Univariate analysis of factors of immunization status

The basic information and immunization status of children with available vaccination data were included for univariate analysis. For NIP vaccine, there were no statistically significant differences in gender and location (*P* > 0.05), but there were statistically significant differences in age interval and tumor diagnosis time (*P*< 0.05). For the first dose of HepB vaccine(χ^2^ = 7.44, *P* =0.027), the second and fourth doses of DTaP vaccine(χ^2^ = 6.16, *P* =0.042), the second dose of MPSV-AC vaccine (χ^2^ = 11.17, *P* = 0.003), HepA-L(χ^2^ = 6.41, *P* =0.031) and HepA-I vaccine(χ^2^ = 5.90, *P*=0.039), the differences in Disease Classification were statistically significant (**Supplementary Tables 1**-**13**).

### 3.5 Multivariate analysis of factors of immunization status

The basic information and immunization status of children with available vaccination data were included to construct a multi-factor ordered logistics regression equation. The results showed that among 394 children with available vaccination data, the vaccination rate of NIP vaccine was lower with the age of older children than those who were less than 5 years old. Compared with the time of diagnosis less than 36 months, the later the diagnosis time, the higher the vaccination rate. For the second dose of MPSV-AC vaccine, the vaccination rate of NB children with high malignant degree was lower than that of GN children with mild disease (**Tables 5**, **6**).

**Table 5:** Multivariate analysis of different age groups.

NIP vaccines	Age(year)						
	1-5*	6-10	*P*	11-15	*P*	16-	*P*
		OR(95%Cl)		OR(95%Cl)		OR(95%Cl)	
HepB_1_	1	0.82(0.07,9.59)	0.006	0.16(0.01,2.17)	0.000	0.01(0.00,0.26)	0.004
HepB_2_	1	1.00(0.21,4.73)	0.992	0.27(0.04,1.71)	0.166	0.02(0.00,0.19)	0.001
HepB_3_	1	2.01(0.88,5.00)	0.095	0.66(0.19,2.25)	0.507	0.13(0.02,0.93)	0.043
BCG	1	1.09(0.36,3.35)	0.876	0.35(0.08,1.49)	0.155	0.04(0.00,0.31)	0.002
IPV_1_	1	1.39(0.37,5.19)	0.623	0.33(0.07,1.76)	0.197	0.02(0.00,0.21)	0.001
IPV_2_	1	1.41(0.49,4.00)	0.516	0.59(0.14,2.47)	0.460	0.04(0.00,0.35)	0.004
bOPV_1_	1	1.82(0.45,7.35)	0.399	0.39(0.07,2.09)	0.272	0.02(0.00,0.25)	0.002
bOPV_2_	1	1.65(0.57,4.86)	0.357	0.63(0.15,2.76)	0.544	0.04(0.00,0.39)	0.005
DTaP_1_	1	1.13(0.36,3.49)	0.835	0.65(0.14,3.05)	0.587	0.05(0.00,0.45)	0.008
DTaP_2_	1	1.37(0.49,3.82)	0.553	0.74(0.17,3.22)	0.688	0.06(0.00,0.54)	0.011
DTaP_3_	1	1.72(0.74,3.98)	0.206	1.09(0.28,4.27)	0.894	0.07(0.00,0.55)	0.011
DTaP_4_	1	1.60(0.91,2.83)	0.104	1.76(0.57,5.47)	0.323	0.14(0.02,0.92)	0.041
MMR_1_	1	3.03(1.43,6.44)	0.004	1.18(0.35,3.94)	0.786	0.19(0.02,1.61)	0.130
MMR_2_	1	2.43(1.37,4.30)	0.002	1.82(0.60,5.48)	0.290	0.10(0.02,0.69)	0.020
JE-L_1_	1	2.14(1.05,4.35)	0.036	1.31(0.38,4.53)	0.670	0.09(0.01,0.72)	0.023
JE-L_2_	1	2.38(1.39,4.05)	0.001	1.99(0.73,5.41)	0.176	0.18(0.03,1.16)	0.072
JE-I_1_	1	2.17(1.04,4.49)	0.038	1.27(0.36,4.46)	0.701	0.08(0.11,0.67)	0.018
JE-I_2_	1	2.29(1.11,4.71)	0.025	1.35(0.38,4.67)	0.640	0.09(0.01,0.69)	0.021
JE-I_3_	1	2.17(1.27,3.70)	0.004	1.76(0.65,4.84)	0.267	0.16(0.03,0.99)	0.049
MPSV-A_1_	1	1.37(0.69,2.72)	0.373	0.88(0.29,2.74)	0.831	0.05(0.01,0.33)	0.002
MPSV-AC_1_	1	1.67(0.98,2.83)	0.060	1.88(0.73,4.93)	0.194	0.12(0.02,0.75)	0.024
HepA-L	1	1.61(0.91,284)	0.103	0.88(0.31,2.48)	0.811	0.02(0.00,0.17)	0.000
HepA-I_1_	1	1.65(0.93,2.91)	0.088	0.84(0.29,2.38)	0.736	0.02(0.00,0.17)	0.000
HepA-I_2_	1	1.76(1.02,3.07)	0.044	0.69(0.26,1.86)	0.461	0.03(0.00,0.20)	0.000

HepB, Hepatitis B vaccine; BCG, Bacillus Calmett-Guerin vaccine; IPV, Inactivated polio vaccine; bOPV, Oral live attenuated polio vaccine; DTaP, Acellular pertussis diphtheria tetanus vaccine; DT, Diphtheria tetanus vaccine; MMR, Measles, mumps, rubella vaccine; JE-L, Live attenuated Japanese encephalitis vaccine; JE-I, Inactivated Japanese encephalitis vaccine; MPSV-A, Meningococcal meningitis-A vaccine; MPSV-AC, Meningococcal meningitis-AC vaccine; HepA-L, Live attenuated Hepatitis A vaccine; HepA-I, Inactivated Hepatitis A vaccine.

*Reference group: according to the age distribution, the actual age was analyzed as a group of 5 years.

**Table 6:** Multivariate analysis of different tumor diagnosis time.

NIP vaccines	tumor diagnosis age(month)						
	0-36*	37-72	*P*	73-108	*P*	109-	*P*
		OR(95%Cl)		OR(95%Cl)		OR(95%Cl)	
DTaP_4_	1	4.15(1.86,9.26)	0.001	3.47(0.95,12.64)	0.060	1.71(0.34,8.72)	0.518
DT	1	1.03(0.55,1.88)	0.933	16.88(5.23,54.56)	0.000	10.69(2.16,52.76)	0.004
MMR_2_	1	5.26(2.24,12.34)	0.000	4.82(1.21,19.27)	0.026	3.97(0.69,22.91)	0.123
JE-L_2_	1	4.64(2.31,9.28)	0.000	8.22(2.03,33.24)	0.003	4.33(0.86,2.94)	0.077
JE-I_3_	1	5.28(2.59,10.76)	0.000	9.36(2.29,38.33)	0.002	4.94(0.97,25.08)	0.054
JE-I_4_	1	4.76(2.51,9.04)	0.000	7.36(2.21,24.53)	0.000	3.01(0.69,13.16)	0.143
MPSV-A_2_	1	3.67(1.69,7.93)	0.001	4.28(1.12,16.31)	0.033	2.94(0.48,18.07)	0.244
MPSV-AC_1_	1	6.48(3.45,12.20)	0.000	14.29(3.94,51.76)	0.000	13.24(2.32,75.29)	0.004
MPSV-AC_2_	1	1.19(0.63,2.27)	0.595	18.02(5.94,54.62)	0.000	9.43(2.01,44,17)	0.004
HepA-L	1	3.96(1.83,8.55)	0.000	12.52(2.12,73.83)	0.005	10.33(1.31,81.67)	0.027
HepA-I_1_	1	4.33(1.96,9.69)	0.000	12.46(2.11,73.73)	0.005	10.59(1.33,84.49)	0.026
HepA-I_2_	1	4.73(2.21,10.07)	0.000	14.55(2.77,76.43)	0.000	7.74(1.38,43.49)	0.020

DTaP, Acellular pertussis diphtheria tetanus vaccine; DT, Diphtheria tetanus vaccine; MMR, Measles, mumps, rubella vaccine; JE-L, Live attenuated Japanese encephalitis vaccine; JE-I, Inactivated Japanese encephalitis vaccine; MPSV-A, Meningococcal meningitis-A vaccine; MPSV-AC, Meningococcal meningitis-AC vaccine; HepA-L, Live attenuated Hepatitis A vaccine; HepA-I, Inactivated Hepatitis A vaccine.

*Reference group: The tumor diagnosis age was analyzed as a group of 36 months, for the median age at tumor diagnosis was 34 months.

### 3.6 Abnormal response to vaccination (AEFI)

According to AEFI monitoring and management information system, there were 2 cases of adverse reactions among NIP vaccines, both occurred after diagnosis and treatment, one case occurred in the third dose of DTaP vaccine, which was redness and swelling at the vaccination site 7 hours after vaccination, the other case occurred in the fourth dose of DTaP vaccine with redness and swelling at the site 4 days after inoculation. The incidence of adverse reactions was calculated was 0.23‰(2/8728). Moreover, complications, severe AEFI, and community AEFI did not occur among these patients.

## 4 Discussion

In this study, NB was the main tumor type of pNT children, and its basic age, gender and disease diagnosis time were similar to those in domestic and foreign studies (7). In our study, the vaccination data of some children could not be queried, we found that the vaccination coverage rate of NIP vaccines reached 71.49% before chemotherapy and 37.67% after chemotherapy, which was lower than that of normal children in China (24), especially the vaccination rate after chemotherapy. We also see a gradual decline in vaccination rates for different doses of the same vaccine, and the delay has been found in other studies (33). By analyzing the factors related to vaccination, the result show that the age of children, the time of tumor diagnosis and disease classification were significantly correlated with whether or not children received some of the vaccines.

In our study, we found that before diagnosis, the vaccination rate of pNT children vaccine was not high, which did not meet the requirements of our country (34). Besides, the vaccination rate of booster immunization (multiple vaccinations) of all vaccines was lower than that of basic immunization (primary vaccinations), which was consistent with the findings of CAO related the vaccination rate of Chinese children in 2012 (35). Importantly, the vaccination rate of these patients >7 years old was lower than 80%.The possible reason were that: a) national vaccination requirements in China before 2006 was low; b) many vaccines were not included in our National Immunization Program in early years; c) vaccination records were handwritten and lacked a unified electronic information management system before 2006, which may lead to the loss of some information (36). For the reasons mentioned above, patient who were born before 2006 had a lower vaccination rate.

We found that the older the child, the lower the vaccination rate; the earlier the tumor diagnosis, the lower the vaccination rate; the more severe the disease classification of children, the lower the coverage rate of the second dose of MPSV-AC vaccine, and they are consistent with the current survey results (37, 38). The possible reasons are as follows: Firstly, it is related to the limitation of vaccination age. The recommended age for NIP vaccination is less than three years old in most cases and less than six years old at the latest; Secondly, it is related to the diagnosis of the disease. Parents focus on the treatment of the disease, and their compliance to immunization is reduced. Moreover, due to the lack of medical knowledge, they have certain concerns about the safety of immunization (39); Thirdly, doctors may recommend stopping vaccination after diagnosis because of the risk associated with vaccination (33); Fourthly, according to the immunization strategy for special children (40), live attenuated vaccines are prohibited for children within three months of chemotherapy, so the immunization plan of children is easy to be delayed.

In our study, it is found that 40.60% (160/394) of the patients were restored to immunization with NIP vaccine after treatment, and the median age of restored immunization was 16.00 (6.00,24.00) months. Only 2 cases of AEFI reaction were found, accounting for 0.23‰. Compared with normal children, the incidence of AEFI reaction was similar (41). In addition, a large number of studies have shown that immunization of pNT children is safe and feasible (28, 31, 42), so a series of measures can be taken to restore the immunization of children. Special services have been set up in recent years to provide vaccination advice for children with special needs: A multidisciplinary immunization model was established for children with hematologic tumors in China, and the incidence of AEFI was 5.9% (43), they also revised the guidelines for the children with cancer, including the type, timing, dosage and even immunization procedures (44).

Because of the effect of disease and treatment, the level of autoantibody and immunity of pNT children is greatly reduced (45). And the 5-year survival rate of high-risk children with NB is less than 2%, but the 5-year survival rate of low- and intermediate-risk children reaches 75%-98%; therefore, children with this disease can be effectively prolonged by improving their prognosis (46). Since immune capacity maybe related to prognosis, while the damage/alteration to the immune system caused by the pNT. This also reminds us it may also be helpful to distinguish the categorization of pNT patients into high-risk, medium-risk, and low-risk categories as it relates to immune status (15, 16). So parents, doctors should take a correct view of the condition (36, 47) that children with tumors can be immunized, and we can take actions to improve the level of children’s autoimmune, and children’s prognosis and quality of life.

The key advantage of this study is that there are few studies on immunization coverage in children with pNT. With the increasing awareness of childhood immunization against diseases, more studies are needed to provide valuable data for the relevant departments’ immunization programs. The limitation of this study is that the data of the objects of this study were from the Children’s Hospital Affiliated to Zhejiang University, which was not randomly selected and could not represent all the children in the whole population, and there was selection error. In addition, the data of this study came from a retrospective study in the real world, which was an observational study and the level of evidence was not strong enough.

## 5 Conclusion

The vaccination coverage rate of pNT children is low, especially the vaccination rate after chemotherapy, and the incidence of AEFI is also low. Therefore, in-depth research on the vaccination of children should be carried out. Parents, doctors, the society and the government should also strengthen the awareness of the safety of immunization, orderly guide the immunization of children, and reduce infection.

## Data availability statement

The raw data supporting the conclusions of this article will be made available by the authors, without undue reservation.

## Ethics statement

This project was approved by the Ethics Committee of Hangzhou Center for Disease Control and Prevention (reference number: 2021-18) and the Ethics Committee of the School of Medicine, Children’s Hospital of Zhejiang University (reference number: 2022-IRB-028).

## Author contributions

HS: Conceptualization, Data curation, Formal analysis, Writing – original draft, Writing – review & editing. YX: Conceptualization, Data curation, Formal analysis, Funding acquisition, Methodology, Writing – original draft, Writing – review & editing. YZ: Investigation, Methodology, Project administration, Resources, Writing – original draft, Writing – review & editing. YL: Software, Supervision, Validation, Visualization, Writing – original draft, Writing – review & editing. XZ: Investigation, Methodology, Project administration, Resources, Writing – original draft, Writing – review & editing. ML: Software, Supervision, Validation, Visualization, Writing – original draft, Writing – review & editing. CJ: Software, Supervision, Validation, Visualization, Writing – original draft, Writing – review & editing.
