# Supplementary material for: Current vaccination status and safety of children with peripheral neuroblastoma in the real-world
Source: Front Immunol. 2024 Jan 8;14:1278258. doi: 10.3389/fimmu.2023.1278258 (PMC10800462; doi:10.3389/fimmu.2023.1278258)
Supplement: Supplementary file 1 [file Table_1.docx]

Supplementary table 1. Analysis of single factor influencing factors of the HepB vaccine

|  | HepB_1_ | | | HepB_2_ | | | HepB_3_ | | |
| --- | --- | --- | --- | --- | --- | --- | --- | --- | --- |
| Characteristics | N（%） | *X^2^* | *p* | N（%） | *X^2^* | *p* | N（%） | *X^2^* | *p* |
| Gender |  | 0.63 | 0.427 |  | 0.75 | 0.390 |  | 5.29 | 0.021 |
| Male | 198(52.4) |  |  | 198(52.3) |  |  | 180(50.8) |  |  |
| Female | 180(47.6) |  |  | 178(47.7) |  |  | 174(49.2) |  |  |
| Location |  | 0.00 | 0.998 |  | 0.07 | 0.788 |  | 0.80 | 0.371 |
| Hangzhou | 118(31.2) |  |  | 117(31.4) |  |  | 113(31.9) |  |  |
| non-Hangzhou | 260(68.8) |  |  | 256(68.6) |  |  | 241(68.1) |  |  |
| Actual Age (year) |  | 58.72 | <0.001 |  | 28.39 | <0.001 |  | 17.85 | <0.001 |
| 1-5 | 106(28.0) |  |  | 104(27.9) |  |  | 93(26.3) |  |  |
| 6-10 | 210(55.6) |  |  | 208(55.8) |  |  | 202(57.1) |  |  |
| 11-15 | 56(14.8) |  |  | 56(15.0) |  |  | 53(15.0) |  |  |
| 16- | 6(1.6) |  |  | 5(1.3) |  |  | 6(1.7) |  |  |
| Tumor diagnosis age (month) |  | 20.68 | <0.001 |  | 16.72 | <0.001 |  | 7.82 | 0.040 |
| 0-36 | 214(56.6) |  |  | 210(56.3) |  |  | 194(54.8) |  |  |
| 37-72 | 113(29.9) |  |  | 113(30.3) |  |  | 111(31.4) |  |  |
| 73-108 | 35(9.3) |  |  | 35(9.4) |  |  | 34(9.6) |  |  |
| 109- | 16(4.2) |  |  | 15(4.0) |  |  | 15(4.2) |  |  |
| Pathological classification |  | 7.44 | 0.027 |  | 4.15 | 0.111 |  | 0.16 | 0.909 |
| NB | 314(83.1) |  |  | 309(82.8) |  |  | 290(81.9) |  |  |
| GNBi | 58(15.3) |  |  | 58(15.5) |  |  | 58(16.4) |  |  |
| GNBn | 0(0.0) |  |  | 0(0.0) |  |  | 0(0.0) |  |  |
| GN | 6(1.6) |  |  | 6(1.6) |  |  | 6(1.7) |  |  |

Abbreviations:NB:neuroblastoma; GNBi:ganglio-neuroblastomainter mixed; GN: ganglioneuroma; GNBn: ganglio-neuroblastomanodular;HepB：hepatitis B vaccine;

Supplementary table 2. Analysis of single factor influencing factors of the BCG vaccine

|  | BCG | | |
| --- | --- | --- | --- |
| Characteristics | N（%） | *X^2^* | *p* |
| Gender |  | 2.51 | 0.113 |
| Male | 188(51.6) |  |  |
| Female | 176(48.4) |  |  |
| Location |  | 1.90 | 0.168 |
| Hangzhou | 117(32.1) |  |  |
| non-Hangzhou | 247(67.9) |  |  |
| Actual Age (year) |  | 23.39 | <0.001 |
| 1-5 | 101(27.7) |  |  |
| 6-10 | 204(56.0) |  |  |
| 11-15 | 54(14.8) |  |  |
| 16- | 5(1.4) |  |  |
| Tumor diagnosis age (month) |  | 13.99 | 0.002 |
| 0-36 | 204(56.0) |  |  |
| 37-72 | 111(30.5) |  |  |
| 73-108 | 35(9.6) |  |  |
| 109- | 14(3.8) |  |  |
| Pathological classification |  | 1.19 | 0.573 |
| NB | 300(82.4) |  |  |
| GNBi | 58(15.9) |  |  |
| GNBn | 0(0.0) |  |  |
| GN | 6(1.6) |  |  |

Abbreviations:NB:neuroblastoma; GNBi:ganglio-neuroblastomainter mixed; GN: ganglioneuroma; GNBn: ganglio-neuroblastomanodular; BCG: Bacillus Calmette Guerinvaccine vaccine;

Supplementary table 3. Analysis of single factor influencing factors of the IPV vaccine

|  | IPV_1_ | | | IPV_2_ | | |
| --- | --- | --- | --- | --- | --- | --- |
| Characteristics | N（%） | *X^2^* | *p* | N（%） | *X^2^* | *p* |
| Gender |  | 0.14 | 0.712 |  | 1.45 | 0.229 |
| Male | 195(52.6) |  |  | 189(51.9) |  |  |
| Female | 176(47.4) |  |  | 175(48.1) |  |  |
| Location |  | 0.30 | 0.584 |  | 0.02 | 0.881 |
| Hangzhou | 117(31.5) |  |  | 114(31.3) |  |  |
| non-Hangzhou | 254(68.5) |  |  | 250(68.7) |  |  |
| Actual Age (year) |  | 26.79 | <0.001 |  | 21.02 | <0.001 |
| 1-5 | 102(27.5) |  |  | 99(27.2) |  |  |
| 6-10 | 208(56.1) |  |  | 204(56.0) |  |  |
| 11-15 | 56(15.1) |  |  | 56(15.4) |  |  |
| 16- | 5(1.3) |  |  | 5(1.4) |  |  |
| Tumor diagnosis age (month) |  | 13.98 | 0.002 |  | 9.94 | 0.014 |
| 0-36 | 207(55.8) |  |  | 202(55.5) |  |  |
| 37-72 | 114(30.7) |  |  | 112(30.8) |  |  |
| 73-108 | 35(9.4) |  |  | 35(9.6) |  |  |
| 109- | 15(4.0) |  |  | 15(4.1) |  |  |
| Pathological classification |  | 3.24 | 0.161 |  | 1.19 | 0.573 |
| NB | 307(82.7) |  |  | 300(82.4) |  |  |
| GNBi | 58(15.6) |  |  | 58(15.9) |  |  |
| GNBn | 0(0.0) |  |  | 0(0.0) |  |  |
| GN | 6(1.6) |  |  | 6(1.6) |  |  |

Abbreviations:NB:neuroblastoma; GNBi:ganglio-neuroblastomainter mixed; GN: ganglioneuroma; GNBn: ganglio-neuroblastomanodular; IPV:inactivated poliomyelitis vaccine;

Supplementary table 4. Analysis of single factor influencing factors of the bOPV vaccine

|  | bOPV_1_ | | |  | bOPV_2_ | | |
| --- | --- | --- | --- | --- | --- | --- | --- |
| Characteristics | N（%） | *X^2^* | *p* | | N（%） | *X^2^* | *p* |
| Gender |  | 0.37 | 0.542 | |  | 2.03 | 0.179 |
| Male | 195(52.4) |  |  | | 189(51.8) |  |  |
| Female | 177(47.6) |  |  | | 176(48.2) |  |  |
| Location |  | 0.17 | 0.681 | |  | 0.00 | 1.000 |
| Hangzhou | 117(31.5) |  |  | | 114(31.2) |  |  |
| non-Hangzhou | 255(68.5) |  |  | | 252(68.8) |  |  |
| Actual Age (year) |  | 28.46 | <0.001 | |  | 22.16 | <0.001 |
| 1-5 | 102(27.4) |  |  | | 99(27.1) |  |  |
| 6-10 | 209(56.2) |  |  | | 205(56.2) |  |  |
| 11-15 | 56(15.1) |  |  | | 56(15.3) |  |  |
| 16- | 5(1.3) |  |  | | 5(1.4) |  |  |
| Tumor diagnosis age (month) |  | 14.81 | 0.001 | |  | 10.39 | 0.011 |
| 0-36 | 208(55.9) |  |  | | 203(55.6) |  |  |
| 37-72 | 114(30.6) |  |  | | 112(30.7) |  |  |
| 73-108 | 35(9.4) |  |  | | 35(9.6) |  |  |
| 109- | 15(4.0) |  |  | | 15(4.1) |  |  |
| Pathological classification |  | 3.67 | 0.147 | |  | 1.40 | 0.558 |
| NB | 308(82.8) |  |  | | 301(82.5) |  |  |
| GNBi | 58(15.6) |  |  | | 58(15.9) |  |  |
| GNBn | 0(0.0) |  |  | | 0(0.0) |  |  |
| GN | 6(1.6) |  |  | | 6(1.6) |  |  |

Abbreviations:NB:neuroblastoma; GNBi:ganglio-neuroblastomainter mixed; GN: ganglioneuroma; GNBn: ganglio-neuroblastomanodular; bOPV：Live Attenuated Oral Poliomyelitis Vaccine;

Supplementary table 5. Analysis of single factor influencing factors of the DTaP vaccine

|  | DTaP_1_ | | | DTaP_2_ | | | DTaP_3_ | | | DTaP_4_ | | |
| --- | --- | --- | --- | --- | --- | --- | --- | --- | --- | --- | --- | --- |
| Characteristics | N（%） | *X^2^* | *p* | N（%） | *X^2^* | *p* | N（%） | *X^2^* | *p* | N（%） | *X^2^* | *p* |
| Gender |  | 1.08 | 0.298 |  | 3.02 | 0.082 |  | 1.23 | 0.267 |  | 1.80 | 0.179 |
| Male | 190(52.1) |  |  | 187(51.5) |  |  | 183(51.8) |  |  | 156(51.0) |  |  |
| Female | 175(47.9) |  |  | 176(48.5) |  |  | 170(48.2) |  |  | 150(49.0) |  |  |
| Location |  | 0.00 | 0.982 |  | 0.02 | 0.896 |  | 0.08 | 0.776 |  | 0.42 | 0.519 |
| Hangzhou | 114(31.2) |  |  | 113(31.1) |  |  | 111(31.4) |  |  | 98(32.0) |  |  |
| non-Hangzhou | 251(68.8) |  |  | 250(68.9) |  |  | 242(68.6) |  |  | 208(68.0) |  |  |
| Actual Age (year) |  | 20.66 | <0.001 |  | 20.01 | <0.001 |  | 23.56 | <0.001 |  | 25.22 | <0.001 |
| 1-5 | 101(27.7) |  |  | 99(27.3) |  |  | 91(26.3) |  |  | 70(22.9) |  |  |
| 6-10 | 203(55.6) |  |  | 203(55.9) |  |  | 200(56.7) |  |  | 177(57.8) |  |  |
| 11-15 | 56(15.3) |  |  | 56(15.4) |  |  | 56(15.9) |  |  | 55(18.0) |  |  |
| 16- | 5(1.4) |  |  | 5(1.4) |  |  | 4(1.1) |  |  | 4(1.3) |  |  |
| Tumor diagnosis age (month) |  | 11.95 | 0.005 |  | 10.44 | 0.010 |  | 11.56 | 0.007 |  | 30.11 | <0.001 |
| 0-36 | 205(56.2) |  |  | 202(55.6) |  |  | 193(54.7) |  |  | 149(48.7) |  |  |
| 37-72 | 111(30.4) |  |  | 112(30.9) |  |  | 112(31.7) |  |  | 110(35.9) |  |  |
| 73-108 | 34(9.3) |  |  | 34(9.4) |  |  | 34(9.6) |  |  | 33(10.8) |  |  |
| 109- | 15(4.1) |  |  | 15(4.1) |  |  | 14(4.0) |  |  | 14(4.6) |  |  |
| Pathological classification |  | 2.67 | 0.229 |  | 1.00 | 0.590 |  | 0.14 | 1.000 |  | 6.16 | 0.042 |
| NB | 302(82.7) |  |  | 299(82.4) |  |  | 289(81.9) |  |  | 243(79.4) |  |  |
| GNBi | 57(15.6) |  |  | 58(16.0) |  |  | 58(16.4) |  |  | 57(18.6) |  |  |
| GNBn | 0(0.0) |  |  | 0(0.0) |  |  | 0(0.0) |  |  | 0(0.0) |  |  |
| GN | 6(1.6) |  |  | 6(1.7) |  |  | 6(1.7) |  |  | 6(2.0) |  |  |

Abbreviations:NB:neuroblastoma; GNBi:ganglio-neuroblastomainter mixed; GN: ganglioneuroma; GNBn: ganglio-neuroblastomanodular; DTaP:Diphtheria and tetanustoxoid with acellular pertussis vaccine;

Supplementary table 6. Analysis of single factor influencing factors of the MMR vaccine

|  | MMR_1_ | | | MMR_2_ | | |
| --- | --- | --- | --- | --- | --- | --- |
| Characteristics | N（%） | *X^2^* | *p* | N（%） | *X^2^* | *p* |
| Gender |  | 0.43 | 0.514 |  | 0.36 | 0.550 |
| Male | 180(52.2) |  |  | 51(49.5) |  |  |
| Female | 165(47.8) |  |  | 52(50.5) |  |  |
| Location |  | 0.18 | 0.669 |  | 0.29 | 0.587 |
| Hangzhou | 109(31.6) |  |  | 34(33.0) |  |  |
| non-Hangzhou | 236(68.4) |  |  | 69(67.0) |  |  |
| Actual Age (year) |  | 22.50 | <0.001 |  | 39.58 | <0.001 |
| 1-5 | 83(24.1) |  |  | 0(0.0) |  |  |
| 6-10 | 200(58.0) |  |  | 65(63.1) |  |  |
| 11-15 | 55(15.9) |  |  | 37(35.9) |  |  |
| 16- | 7(2.0) |  |  | 1(1.0) |  |  |
| Tumor diagnosis age (month) |  | 9.99 | 0.015 |  | 35.51 | <0.001 |
| 0-36 | 182(52.8) |  |  | 36(19.5) |  |  |
| 37-72 | 111(32.2) |  |  | 37(30.3) |  |  |
| 73-108 | 36(10.4) |  |  | 33(17) |  |  |
| 109- | 16(4.6) |  |  | 16(13.1) |  |  |
| Pathological classification |  | 0.23 | 1.000 |  | 2.91 | 0.205 |
| NB | 282(81.7) |  |  | 74(71.8) |  |  |
| GNBi | 57(16.5) |  |  | 25(24.3) |  |  |
| GNBn | 0(0.0) |  |  | 0(0.0) |  |  |
| GN | 6(1.7) |  |  | 4(3.9) |  |  |

Abbreviations:NB:neuroblastoma; GNBi:ganglio-neuroblastomainter mixed; GN: ganglioneuroma; GNBn: ganglio-neuroblastomanodular; MMR：Measles mumps and rubella vaccine;

Supplementary table 7. Analysis of single factor influencing factors of the JE-L vaccine

|  | JE-L_1_ | | | JE-L_2_ | | |
| --- | --- | --- | --- | --- | --- | --- |
| Characteristics | N（%） | *X^2^* | *p* | N（%） | *X^2^* | *p* |
| Gender |  | 1.41 | 0.234 |  | 0.00 | 0.969 |
| Male | 176(51.6) |  |  | 145(52.7) |  |  |
| Female | 165(48.4) |  |  | 130(47.3) |  |  |
| Location |  | 0.66 | 0.417 |  | 0.56 | 0.456 |
| Hangzhou | 109(32.0) |  |  | 89(32.4) |  |  |
| non-Hangzhou | 232(68.0) |  |  | 186(67.6) |  |  |
| Actual Age (year) |  | 22.16 | <0.001 |  | 39.94 | <0.001 |
| 1-5 | 84(24.6) |  |  | 51(18.5) |  |  |
| 6-10 | 196(57.5) |  |  | 166(60.4) |  |  |
| 11-15 | 56(16.4) |  |  | 53(19.3) |  |  |
| 16- | 5(1.5) |  |  | 5(1.8) |  |  |
| Tumor diagnosis age (month) |  | 11.52 | 0.007 |  | 45.65 | <0.001 |
| 0-36 | 180(52.8) |  |  | 121(44.0) |  |  |
| 37-72 | 111(32.6) |  |  | 105(38.2) |  |  |
| 73-108 | 35(10.3) |  |  | 34(12.4) |  |  |
| 109- | 15(4.4) |  |  | 15(5.5) |  |  |
| Pathological classification |  | 0.37 | 0.935 |  | 4.02 | 0.107 |
| NB | 278(81.5) |  |  | 219(79.6) |  |  |
| GNBi | 57(16.7) |  |  | 52(18.9) |  |  |
| GNBn | 0(0.0) |  |  | 0(0.0) |  |  |
| GN | 6(1.8) |  |  | 4(1.5) |  |  |

Abbreviations:NB:neuroblastoma; GNBi:ganglio-neuroblastomainter mixed; GN: ganglioneuroma; GNBn: ganglio-neuroblastomanodular; JE-L：Live attenuated Japanese encephalitis vaccine;

Supplementary table 8. Analysis of single factor influencing factors of the JE-I vaccine

|  | JE-I_1_ | | | JE-I_2_ | | | JE-I3 | | | JE-I4 | | |
| --- | --- | --- | --- | --- | --- | --- | --- | --- | --- | --- | --- | --- |
| Characteristics | N（%） | *X^2^* | *p* | N（%） | *X^2^* | *p* | N（%） | *X^2^* | *p* | N（%） | *X^2^* | *p* |
| Gender |  | 0.86 | 0.355 |  | 1.12 | 0.290 |  | 0.14 | 0.707 |  | 0.23 | 0.640 |
| Male | 178(51.9) |  |  | 177(51.8) |  |  | 144(52.2) |  |  | 134(51.9) |  |  |
| Female | 165(48.1) |  |  | 165(48.2) |  |  | 132(47.8) |  |  | 124(48.1) |  |  |
| Location |  | 0.89 | 0.344 |  | 1.08 | 0.299 |  | 1.32 | 0.251 |  | 0.11 | 0.739 |
| Hangzhou | 110(32.1) |  |  | 110(32.2) |  |  | 91(33.0) |  |  | 82(31.8) |  |  |
| non-Hangzhou | 233(67.9) |  |  | 232(67.8) |  |  | 185(67.0) |  |  | 176(68.2) |  |  |
| Actual Age (year) |  | 22.16 | <0.001 |  | 23.27 | <0.001 |  | 38.13 | <0.001 |  | 30.25 | <0.001 |
| 1-5 | 85(24.8) |  |  | 84(24.6) |  |  | 52(18.8) |  |  | 50(19.4) |  |  |
| 6-10 | 197(57.4) |  |  | 197(57.6) |  |  | 166(60.1) |  |  | 151(58.5) |  |  |
| 11-15 | 56(16.3) |  |  | 56(16.4) |  |  | 53(19.2) |  |  | 52(20.2) |  |  |
| 16- | 5(1.5) |  |  | 5(1.5) |  |  | 5(1.8) |  |  | 5(1.9) |  |  |
| Tumor diagnosis age (month) |  | 10.65 | 0.011 |  | 11.07 | 0.009 |  | 47.66 | <0.001 |  | 47.73 | <0.001 |
| 0-36 | 182(53.1) |  |  | 181(52.9) |  |  | 131(43.8) |  |  | 110(42.6) |  |  |
| 37-72 | 111(32.4) |  |  | 111(32.5) |  |  | 106(38.4) |  |  | 101(39.1) |  |  |
| 73-108 | 35(10.2) |  |  | 35(10.2) |  |  | 34(12.3) |  |  | 33(12.8) |  |  |
| 109- | 15(4.4) |  |  | 15(4.4) |  |  | 15(5.4) |  |  | 14(5.4) |  |  |
| Pathological classification |  | 0.34 | 0.931 |  | 0.33 | 0.934 |  | 3.06 | 0.202 |  | 2.35 | 0.334 |
| NB | 281(81.9) |  |  | 280(81.9) |  |  | 220(79.7) |  |  | 206(79.8) |  |  |
| GNBi | 56(16.3) |  |  | 56(16.4) |  |  | 51(18.5) |  |  | 47(18.2) |  |  |
| GNBn | 0(0.0) |  |  | 0(0.0) |  |  | 0(0.0) |  |  | 0(0.0) |  |  |
| GN | 6(1.7) |  |  | 6(1.8) |  |  | 5(1.8) |  |  | 5(1.9) |  |  |

Abbreviations:NB:neuroblastoma; GNBi:ganglio-neuroblastomainter mixed; GN: ganglioneuroma; GNBn: ganglio-neuroblastomanodular; JE-I, Inactivated Japanese Encephalitis vaccine;

Supplementary table 9. Analysis of single factor influencing factors of the MPSV-A vaccine

|  | MPSV-A_1_ | | | MPSV-A_2_ | | |
| --- | --- | --- | --- | --- | --- | --- |
| Characteristics | N（%） | *X^2^* | *p* | N（%） | *X^2^* | *p* |
| Gender |  | 0.14 | 0.710 |  | 0.000 | 0.986 |
| Male | 175(52.4) |  |  | 162(52.8) |  |  |
| Female | 159(47.6) |  |  | 145(47.2) |  |  |
| Location |  | 0.01 | 0.935 |  | 0.233 | 0.630 |
| Hangzhou | 104(31.1) |  |  | 94(30.6) |  |  |
| non-Hangzhou | 230(68.9) |  |  | 213(69.4) |  |  |
| Actual Age (year) |  | 22.61 | <0.001 |  | 37.807 | <0.001 |
| 1-5 | 87(26.0) |  |  | 77(25.1) |  |  |
| 6-10 | 190(56.9) |  |  | 177(57.7) |  |  |
| 11-15 | 54(16.2) |  |  | 53(17.3) |  |  |
| 16- | 3(0.9) |  |  | 0(0) |  |  |
| Tumor diagnosis age (month) |  | 10.05 | 0.016 |  | 22.161 | <0.001 |
| 0-36 | 179(53.6) |  |  | 156(50.8) |  |  |
| 37-72 | 109(32.6) |  |  | 108(35.2) |  |  |
| 73-108 | 32(9.6) |  |  | 31(10.1) |  |  |
| 109- | 14(4.2) |  |  | 12(3.9) |  |  |
| Pathological classification |  | 0.93 | 0.620 |  | 3.328 | 0.152 |
| NB | 271(81.1) |  |  | 246(80.1) |  |  |
| GNBi | 57(17.1) |  |  | 55(17.9) |  |  |
| GNBn | 0(0.0) |  |  | 0(0.0) |  |  |
| GN | 6(1.8) |  |  | 6(2.0) |  |  |

Abbreviations:NB:neuroblastoma; GNBi:ganglio-neuroblastomainter mixed; GN: ganglioneuroma; GNBn: ganglio-neuroblastomanodular; MPSV-A, Meningococcal polysaccharide vaccineA;

Supplementary table 10. Analysis of single factor influencing factors of the MPSV-AC vaccine

|  | MPSV-AC_1_ | | | MPSV-AC_2_ | | |
| --- | --- | --- | --- | --- | --- | --- |
| Characteristics | N（%） | *X^2^* | *p* | N（%） | *X^2^* | *p* |
| Gender |  | 0.05 | 0.818 |  | 0.60 | 0.438 |
| Male | 124(52.3) |  |  | 51(49.5) |  |  |
| Female | 113(47.7) |  |  | 52(50.5) |  |  |
| Location |  | 3.17 | 0.075 |  | 0.21 | 0.648 |
| Hangzhou | 82(34.6) |  |  | 34(33.0) |  |  |
| non-Hangzhou | 155(65.4) |  |  | 69(67.0) |  |  |
| Actual Age (year) |  | 41.93 | <0.001 |  | 94.40 | <0.001 |
| 1-5 | 40(16.9) |  |  | 0(0) |  |  |
| 6-10 | 140(59.1) |  |  | 65(63.1) |  |  |
| 11-15 | 52(21.9) |  |  | 37(35.9) |  |  |
| 16- | 5(2.1) |  |  | 1(1.0) |  |  |
| Tumor diagnosis age (month) |  | 75.26 | <0.001 |  | 84.92 | <0.001 |
| 0-36 | 89(37.6) |  |  | 28(27.2) |  |  |
| 37-72 | 99(41.8) |  |  | 33(32.0) |  |  |
| 73-108 | 33(13.9) |  |  | 30(29.1) |  |  |
| 109- | 16(6.8) |  |  | 12(11.7) |  |  |
| Pathological classification |  | 4.04 | 0.137 |  | 11.17 | 0.003 |
| NB | 187(78.9) |  |  | 74(71.8) |  |  |
| GNBi | 45(19.0) |  |  | 25(24.3) |  |  |
| GNBn | 0(0.0) |  |  | 0(0.0) |  |  |
| GN | 5(2.1) |  |  | 4(3.9) |  |  |

Abbreviations:NB:neuroblastoma; GNBi:ganglio-neuroblastomainter mixed; GN: ganglioneuroma; GNBn: ganglio-neuroblastomanodular; MPSV-AC: Meningococcal polysaccharide vaccineAC;

Supplementary table 11. Analysis of single factor influencing factors of the HeA-L vaccines

|  | HepA-L | | |
| --- | --- | --- | --- |
| Characteristics | N(%) | *X^2^* | *p* |
| Gender |  | 0.94 | 0.333 |
| Male | 153(52.0) |  |  |
| Female | 141(48.0) |  |  |
| Location |  | 1.55 | 0.214 |
| Hangzhou | 96(32.7) |  |  |
| non-Hangzhou | 198(67.3) |  |  |
| Actual Age (year) |  | 28.33 | <0.001 |
| 1-5 | 70(23.0) |  |  |
| 6-10 | 178(58.4) |  |  |
| 11-15 | 54(17.7) |  |  |
| 16- | 3(1.0) |  |  |
| Tumor diagnosis age (month) |  | 26.97 | <0.001 |
| 0-36 | 148(48.5) |  |  |
| 37-72 | 108(35.4) |  |  |
| 73-108 | 34(11.1) |  |  |
| 109- | 15(4.9) |  |  |
| Pathological classification |  | 6.41 | 0.031 |
| NB | 242(79.3) |  |  |
| GNBi | 57(18.7) |  |  |
| GNBn | 0(0.0) |  |  |
| GN | 6(2.0) |  |  |

Abbreviations:NB:neuroblastoma; GNBi:ganglio-neuroblastomainter mixed; GN: ganglioneuroma; GNBn: ganglio-neuroblastomanodular; HepA-L, live attenuated hepatitis A vaccine;

Supplementary table 12. Analysis of single factor influencing factors of the DT vaccines

|  | DT | | |
| --- | --- | --- | --- |
| Characteristics | N(%) | *X^2^* | *p* |
| Gender |  | 1.39 | 0.238 |
| Male | 59(48.4) |  |  |
| Female | 63(51.6) |  |  |
| Location |  | 1.33 | 0.248 |
| Hangzhou | 43(35.2) |  |  |
| non-Hangzhou | 79(64.8) |  |  |
| Actual Age (year) |  | 111.20 | <0.001 |
| 1-5 | 0(0.0) |  |  |
| 6-10 | 75(61.5) |  |  |
| 11-15 | 41(33.6) |  |  |
| 16- | 6(4.9) |  |  |
| Tumor diagnosis age (month) |  | 96.25 | <0.001 |
| 0-36 | 36(29.5) |  |  |
| 37-72 | 37(30.3) |  |  |
| 73-108 | 33(27.0) |  |  |
| 109- | 16(13.1) |  |  |
| Pathological classification |  | 5.44 | 0.049 |
| NB | 92(75.4) |  |  |
| GNBi | 27(22.1) |  |  |
| GNBn | 0(0.0) |  |  |
| GN | 3(2.5) |  |  |

Abbreviations:NB:neuroblastoma; GNBi:ganglio-neuroblastomainter mixed; GN: ganglioneuroma; GNBn: ganglio-neuroblastomanodular; DT: Diphtheria Tetanus vaccine;.

Supplementary table 13. Analysis of single factor influencing factors of the HepA-I vaccine

|  | HepA-I_1_ | | | HepA-I_2_ | | |
| --- | --- | --- | --- | --- | --- | --- |
| Characteristics | N（%） | *X*^2^ | *p* | N（%） | *X*^2^ | *p* |
| Gender |  | 1.21 | 0.271 |  | 0.26 | 0.609 |
| Male | 157(51.5) |  |  | 157(51.3) |  |  |
| Female | 148(48.5) |  |  | 149(48.7) |  |  |
| Location |  | 1.36 | 0.243 |  | 1.11 | 0.292 |
| Hangzhou | 100(32.8) |  |  | 100(32.7) |  |  |
| non-Hangzhou | 205(67.2) |  |  | 206(67.3) |  |  |
| Actual Age (year) |  | 29.09 | <0.001 |  | 28.83 | <0.001 |
| 1-5 | 70(22.9) |  |  | 65(22.1) |  |  |
| 6-10 | 179(58.5) |  |  | 175(59.5) |  |  |
| 11-15 | 54(17.6) |  |  | 51(17.3) |  |  |
| 16- | 3(1.0) |  |  | 3(1.0) |  |  |
| Tumor diagnosis age (month) |  | 29.11 | <0.001 |  | 31.85 | <0.001 |
| 0-36 | 148(48.4) |  |  | 139(47.3) |  |  |
| 37-72 | 109(35.6) |  |  | 107(36.4) |  |  |
| 73-108 | 34(11.1) |  |  | 34(11.6) |  |  |
| 109- | 15(4.9) |  |  | 14(4.8) |  |  |
| Pathological classification |  | 7.92 | 0.015 |  | 5.90 | 0.039 |
| NB | 242(79.1) |  |  | 233(19.0) |  |  |
| GNBi | 58(19.0) |  |  | 56(19.0) |  |  |
| GNBn | 0(0.0) |  |  | 0(0.0) |  |  |
| GN | 6(2.0) |  |  | 5(1.7) |  |  |

Abbreviations:NB:neuroblastoma; GNBi:ganglio-neuroblastomainter mixed; GN: ganglioneuroma; GNBn: ganglio-neuroblastomanodular; HepA-I:Inactivated HepatitisA vaccine.
